# Imaging of nuclear magnetic resonance spin–lattice relaxation activation energy in cartilage

**DOI:** 10.1098/rsos.180221

**Published:** 2018-07-11

**Authors:** R. J. Foster, R. A. Damion, M. E. Ries, S. W. Smye, D. G. McGonagle, D. A. Binks, A. Radjenovic

**Affiliations:** 1 Soft Matter Physics Group, School of Physics and Astronomy, University of Leeds, Leeds LS2 9JT, UK; 2 Leeds Institute of Rheumatic and Musculoskeletal Medicine, University of Leeds, Leeds LS2 9JT, UK; 3 School of Medicine, University of Leeds, Leeds LS2 9JT, UK; 4 Leeds Musculoskeletal Biomedical Research Unit, Chapel Allerton Hospital, University of Leeds, Leeds LS4 7SA, UK

**Keywords:** cartilage, magnetic resonance imaging, activation energy, *T*_1_

## Abstract

Samples of human and bovine cartilage have been examined using magnetic resonance imaging to determine the proton nuclear magnetic resonance spin–lattice relaxation time, *T*_1_, as a function of depth within through the cartilage tissue. *T*_1_ was measured at five to seven temperatures between 8 and 38°C. From this, it is shown that the *T*_1_ relaxation time is well described by Arrhenius-type behaviour and the activation energy of the relaxation process is quantified. The activation energy within the cartilage is approximately 11 ± 2 kJ mol^−1^ with this notably being less than that for both pure water (16.6 ± 0.4 kJ mol^−1^) and the phosphate-buffered solution in which the cartilage was immersed (14.7 ± 1.0 kJ mol^−1^). It is shown that this activation energy increases as a function of depth in the cartilage. It is known that cartilage composition varies with depth, and hence, these results have been interpreted in terms of the structure within the cartilage tissue and the association of the water with the macromolecular constituents of the cartilage.

## 1. Introduction

Articular cartilage is mainly composed of type II collagen, glycosaminoglycans (GAGs) and interstitial water. The distribution of these constituents is heterogeneous and the collagen is also anisotropic. The organization and complex interplay of these components imbues the tissue with beneficial load-bearing and lubrication properties and, because of this, cartilage and its function have been the subject of recent intense research, for example see the reviews in Binks *et al.* [1], Mow & Huiskes [2], Vaca-González *et al.* [3] and Wilson *et al.* [4] and the mini-review series introduced by Pacifici [5] on cartilage biology and pathology. A major motivating factor for the study of articular cartilage is that cartilage is a key site for both the initiation and progression of osteoarthritis (OA), which is a highly prevalent age-related condition for which the treatment for the end stage of the disease is joint replacement [6]. The early stages of OA are associated with changes in the macromolecular composition of the cartilage and softening of the tissue due to increased water content, which is thought to occur prior to tissue loss.

Owing to its non-invasive nature, magnetic resonance imaging (MRI) is an extremely powerful tool for the study of cartilage tissue, as it can probe the properties of the water within the tissue body. Through understanding the interaction of the interstitial water with the macromolecular constituents within the cartilage tissue and the effect of this on MRI relaxation times, indirect information on the extracellular matrix can be determined. The challenge is then to link this information on the structure and composition of the tissue to its biochemical and biomechanical properties.

Quantitative MRI has been used to study cartilage, both *in vitro* [7–10] and *in vivo* [11–14]. Measurements of the spin–spin relaxation time, *T*_2_, have been shown to be sensitive to a wide range of different parameters including collagen concentration [15], fibril orientation [10], fibril organization [16], GAGs [17] and changes in tissue water content [18]. Quantitative mapping of more complex MRI parameters, such as the relaxation time in the rotating reference frame [19,20], relaxation times of sodium nuclei [21–23] and gagCEST [24,25], have also been reported to be sensitive to changes in GAG content within the tissue.

Measurement of the spin–lattice relaxation time, *T*_1_, has often been disregarded because it shows relatively little contrast or variation throughout the cartilage body [26]. *T*_1_ typically exhibits a monotonic decrease through the depth of the cartilage [27]. In addition, long measurement times for *T*_1_ have meant that investigators interested in clinical applications have instead focused on imaging *T*_1_ in the presence of a gadolinium contrast agent, based on the dGEMRIC method [28], because it has been shown that the contrast agent tends to concentrate in regions of lowest GAG content and can therefore be used to show regions of early cartilage degeneration, before the formation of focal lesions [13,29].

However, it has also been shown that *T*_1_ is sensitive to the changes in water content [17,27,30], and therefore, it offers the opportunity to use the interstitial water within cartilage as a probe to study the structure and composition of the cartilage. In the early stages of OA, it has been reported that the water content of cartilage increases because the degeneration disrupts the extracellular matrix. It has therefore been postulated that the measurement of water content can provide useful information on the degree of early cartilage degeneration, and thus, *T*_1_ could provide an effective biomarker in cartilage [27].

The Arrhenius activation energy, *E*_A_, is a widely used experimentally determined parameter that relates the rate of a physical or chemical process to temperature. Within the context of NMR relaxation times, this can be interpreted as an energy barrier for the relaxation process to occur. Spin–lattice relaxation in biological samples results predominately from dipolar interactions between the hydrogen nuclei in water molecules and the interactions of these nuclei with the surrounding constituent macromolecules. The strength of these interactions is determined, in part, by the rotational and translational motion of the water molecules. The temperature dependence of this motion gives rise to the temperature dependence of the relaxation time, *T*_1_ [31]. Activation energies have also been determined in diffusion NMR in *ex vivo* white matter [32] and in muscle tissue [33]. This approach has been used within clinical MRI to monitor tissue temperature in MR thermometry [31,34].

*T*_2_ has also been shown to be sensitive to water content, and an increase in *T*_2_ relaxation time with increasing temperature has been observed. However, in tissues, *T*_2_ values are affected by a wide variety of different factors such as slow molecular tumbling and chemical exchange, resulting in $T_{2}\ll T_{1}$ [21], and these factors complicate the temperature dependence [31]. Indeed, the analysis of proton spin–spin relaxation temperature dependence in muscle tissue resulted in an activation energy that was negative [33].

In this study, we have therefore chosen to investigate the depth-wise change in *T*_1_ relaxation time as a function of temperature to extract the depth dependence of the *E*_A_ for the *T*_1_ relaxation process, in order to determine if new structural information could be highlighted by this approach. We present activation energy profiles for the first time in articular cartilage and suggest a possible explanation for our observations.

## 2. Material and methods

### 2.1. Cartilage samples

Samples of human and bovine cartilage were examined *in vitro* using MRI. Human cartilage from the tibial plateau was obtained post-mortem through the Leeds GIFT2 Research Tissue Project [35] in accordance with ethical approval 10/H1313/48 from the Local Research Ethics Committee (Leeds). Informed consent was obtained from donors' next of kin by NHS Blood and Transplant Tissue Services on behalf of GIFT2, under a Service Level Agreement to their agreed policies and procedures which are NHS Research Ethics Committee approved. This is in line with approved standards for taking consent by the Human Tissue Authority.

Six samples of cartilage from different individual donors were imaged. All donors had no known diagnosis of OA, rheumatoid arthritis or other joint disease, and it has been assumed that although all the donors were in the age range of 65–86 years, the samples were all ‘healthy’ cartilage. Details for the donors can be found in the electronic supplementary material. Samples were procured according to the GIFT2 Research Tissue Project protocols: procurement of samples from donors was achieved within 1–7 days of death, and all donors were kept refrigerated in a standard mortuary after death. Donor knees were kept intact and frozen at −80°C before thawing immediately prior to cartilage harvesting. The samples were then immersed in phosphate-buffered solution (PBS) and imaging was started as soon as possible after harvesting (approx. 2 h).

Bovine legs were obtained from a local abattoir (John Penny and Sons, Leeds, UK) from animals which had been killed as part of the normal food chain following appropriate veterinary inspection. Cartilage was harvested from the patellofemoral groove of healthy 18-month-old cows (within 72 h of slaughter). Permission was obtained from the abattoir to use these animal tissues for research. Samples had been frozen in a standard food freezer at −20°C prior to imaging and were thus defrosted at ambient temperatures before being immersed in PBS for imaging.

Two samples are presented in the main body of the paper: one human and one bovine. Data for the five other human samples can be found in the electronic supplementary material.

### 2.2. Material and methods

#### 2.2.1. MRI protocol

Imaging of the samples was achieved using a progressive saturation sequence at 9.4 T (Bruker AVANCE™ II 400 MHz). For human cartilage, a set of seven repetition times, *T*_R_ (adapted from Nissi *et al.* [26]), were used for all temperatures as given in table 1. The spin-echo time was 14 ms, slice thickness 1 mm and the in-plane resolution was 100 µm pixel^−1^. For bovine cartilage, a set of 15 or 16 repetition times were used which were varied with temperature, as presented in table 1. The spin-echo time was 8.56 ms, slice thickness 2 mm and the in-plane resolution was 70 µm pixel^−1^ (perpendicular to the cartilage surface) by 117 µm pixel^−1^ (parallel to the surface). All signal averaging (table 1) was performed prior to image reconstruction. The number of averages and *T*_R_s were varied at each temperature to obtain similar signal to noise at each temperature.

**Table 1.** Repetition times, *T*_R_, and number of averages used in the progressive saturation sequence for the human and bovine cartilage samples at various temperatures.

sample	temperature (°C)	*T*_R_ (ms)	averages
human	9.1, 12.5, 15.9, 20.0, 25.0, 29.4, 34.5	200, 500, 1000, 1500, 2500, 4000, 8000	6
	8.1	295, 458, 634, 824, 1033, 1263, 1519, 1808, 2139, 2528, 2999, 3595, 4407, 5692, 9000	4
	14.9	100, 287, 487, 703, 937, 1193, 1475, 1790, 2144, 2551, 3029, 3608, 4340, 5340, 6924, 11 040	1
bovine	22.3	213, 440, 684, 946, 1231, 1543, 1886, 2268, 2699, 3195, 3776, 4478, 5368, 6582, 8500, 13 440	1
	30.0	100, 370, 660, 970, 1310, 1680, 2090, 2550, 3060, 3650, 4350, 5190, 6250, 7700, 10 000, 16 000	2
	38.1	622, 966, 1338, 1740, 2181, 2666, 3206, 3816, 4516, 5337, 6331, 7589, 9304, 12 017, 19 000	2

For the other human samples presented in the electronic supplementary material, the number of *T*_R_s chosen and the matrix size were varied to account for variation in physical sample size, to ensure imaging of the sample over a range of temperatures could be obtained before significant sample degradation.

Images were obtained at a range of temperatures from 8 to 38°C. Temperature control was achieved using the water cooling system for the gradient coils on the NMR system. The samples within the imaging coils were allowed a minimum of 1 h to equilibrate with the water temperature before data collection was initiated. The temperature was monitored using a thermocouple within the bore of the magnet. Temperature data were recorded during imaging and the measurement temperature was determined as the average temperature during the imaging sequence. Fluctuations in the monitored temperature were noted to be less than 1°C at all temperatures measured.

#### 2.2.2. *T*_1_ and *E*_A_ profiles

**Figure 1.**
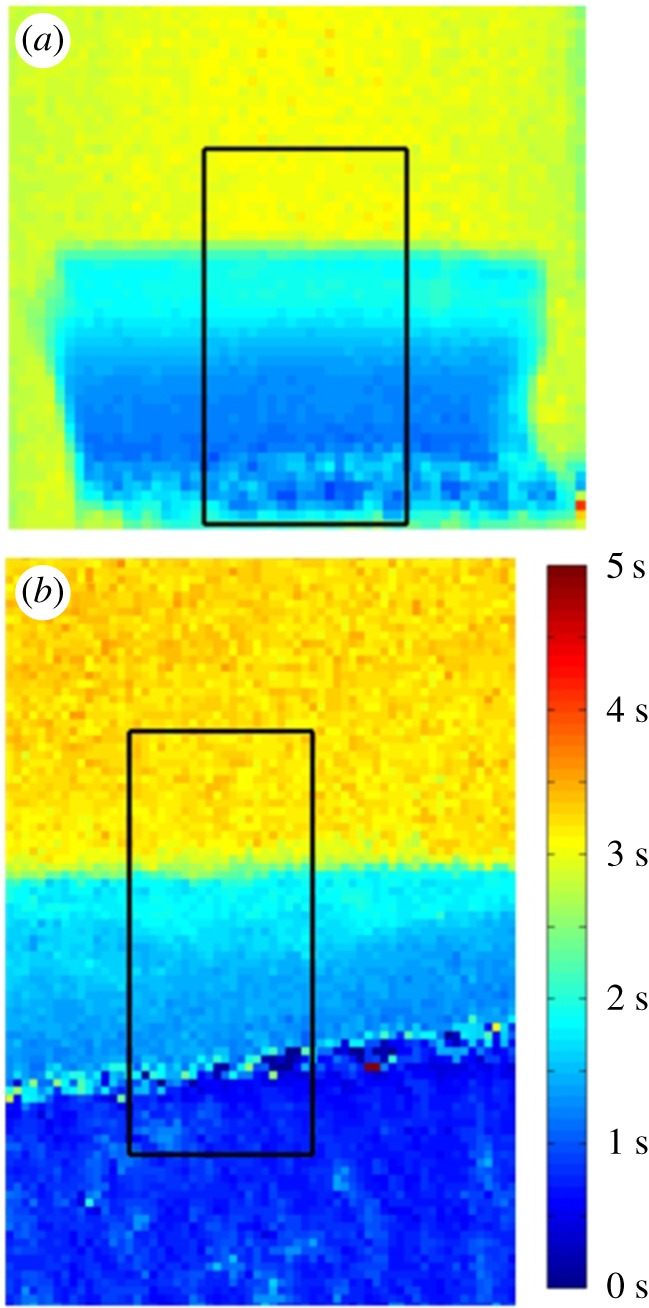
*T*_1_ relaxation time maps of (*a*) bovine cartilage at 8°C and (*b*) human cartilage at 20°C. The rectangle in each image shows the ROI used to generate the *T*_1_ and *E*_A_ profiles.

*T*_1_ relaxation time maps were calculated for each temperature measured, by fitting a mono-exponential function on a pixel-by-pixel basis (Matlab 7.14.0 R2013a, Mathworks, Inc., Natick, MA, USA), which was shown to be a good fit throughout the cartilage tissue, with *r*^2^ > 0.99 in all cases. Example fits can be seen in the electronic supplementary material. *T*_1_ relaxation time profiles were then generated through the depth of the cartilage by choosing a region of interest (ROI) from the *T*_1_ relaxation time maps, as shown in figure 1. Details of code used can be found in the electronic supplementary material.

ROIs were chosen by eye such that they included PBS from above the cartilage surface and at least some portion into the subchondral bone network below the calcified collagen, at the deepest layer of cartilage. This was done to ensure that the full thickness of cartilage was included within the ROI. The ROIs were also chosen such that there was minimal curvature of the cartilage surface.

Average *T*_1_ relaxation time depth-wise profiles were obtained by averaging across each row (parallel to the cartilage surface) within the chosen ROI. Activation energy profiles were obtained by linearly fitting the logarithm of average *T*_1_ relaxation times with the reciprocal of temperature, in accordance with the Arrhenius relationship [31]:

$$\frac{1}{T_{1}}=A\exp\left(\frac{E_{A}}{RT}\right),$$

where *T* is the absolute temperature, *R* is the ideal gas constant and *A* is the pre-exponential factor. This linear fitting was weighted to the uncertainties determined in the fitted *T*_1_ values. *E*_A_ was determined on a pixel-by-pixel basis and then averaged across each row using the same method as that for *T*_1_. The surface of the cartilage was determined from the *T*_1_ relaxation time profiles and was defined as being the midpoint of the change in *T*_1_ relaxation time from the consistent value observed in the PBS to the smooth decrease in *T*_1_ through the main body of cartilage. Scaling of the depth-wise profiles was achieved by using the known in-plane resolution of the images. Details of the code used can be found in the electronic supplementary material.

## 3. Results and discussion

### 3.1. *T*_1_ relaxation time profiles

**Figure 2.**
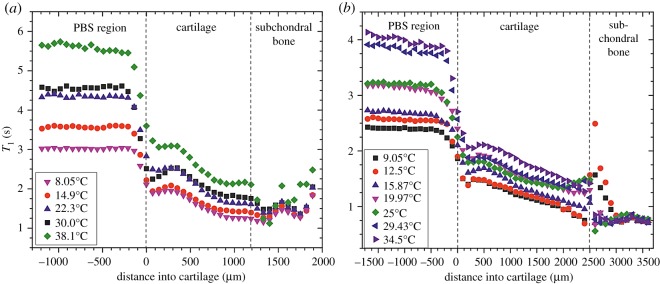
*T*_1_ profiles for the selected ROI as a function of depth through the cartilage samples. (*a*) Bovine and (*b*) human. The legend in each figure panel indicates the temperature at which each *T*_1_ profile was obtained. The lines indicating PBS, cartilage and subchondral bone are drawn to guide the eye. The PBS/cartilage dividing line is taken as the cartilage surface (0 µm), and the cartilage/bone interface is estimated as the point at which there is significant deviation from the smooth decrease in *T*_1_. Note that error bars in *T*_1_ are not displayed as they are smaller than the size of the data points—all errors were less than 1%.

Figure 2 shows the depth-wise *T*_1_ relaxation time profiles obtained for both bovine and human cartilage imaged in this study, for a range of temperatures. In the plots, the cartilage surface is placed at a distance of zero, with the PBS on the left (negative distance values) and the cartilage extending to the right (positive distance values). It can be seen that the *T*_1_ of the cartilage is lower than that in the PBS and exhibits a relatively smooth decrease through the cartilage. At the deepest regions, within the calcified parts, the *T*_1_ relaxation times become noisy due to the decreased signal intensity arising from the low water content at these depths. It can also be seen that, in the PBS and main body of the cartilage, *T*_1_ increases with temperature.

In both cases, human and bovine, the *T*_1_ values are seen to generally increase with increasing temperature. The rate of change of *T*_1_ with temperature (d*T*_1_/d*T*) has been determined in other studies [31]. For the cartilage in this study, this has been found to be in the range of 1.4–1.9%/°C for the human cartilage and 1.5–1.7%/°C for the bovine cartilage. These values are similar to those reported previously for other biological tissues, for example 1.4%/°C in bovine muscle [36], 1–2%/°C in liver [37] and 0.97%/°C in fat [38].

The surface of the cartilage samples was also determined using these *T*_1_ profiles; however, as can be seen in figure 2, there is a gradient between the *T*_1_ values in the PBS and those within the main body of the cartilage. It is believed that this is primarily due to a partial volume average artefact. Thus, the surface was defined to be the midpoint of the slope from where the *T*_1_ values start to decrease to where the values are clearly within the main cartilage body.

The *T*_1_ values in the region identified in figure 2 as subchondral bone are unreliable due to poor signal-to-noise ratio and the complexity of that tissue. Subchondral (cortical) bone consists of various cells embedded in a generally low-porosity matrix but which also possesses larger extracellular spaces such as the Haversian canals. In addition to the intra- and extracellular water contributions to the proton NMR signal, a component is believed to originate from protons of mobile methylene lipids [39]. The situation is potentially further complicated due to the chemical shift between the water and lipid protons, which can displace the lipid signals in the images. Also, depending on the details of the imaging sequence, chemically shifted triglyceride components from the neighbouring trabecular bone can intrude [40]. Therefore, in this region, mono-exponential *T*_1_ fitting is no longer appropriate. However, this region is beyond the scope of investigation in this paper, which has focused on the properties of the cartilage.

Furthermore, one can see in figure 1*b* that the interface between the cartilage and bone is not aligned with the boundaries of the ROI. The effect of this is that the row averaging will introduce a source of error into both the cartilage and bone values in this interfacial region. Although there exist more sophisticated ways of dealing with such situations, for the simple method we employed in this work it was not possible to align both the PBS–cartilage and the cartilage–bone interfaces. Here, we chose to align the PBS–cartilage interface because it is known that the surface zone of cartilage is relatively shallow (and typically thinner than the deep zone), and this area of the cartilage is of particular importance.

*T*_1_ plots for the other samples imaged in this study can be found in the electronic supplementary material. All samples show similar changes in *T*_1_ with depth through the cartilage.

### 3.2. Arrhenius plots

**Figure 3.**
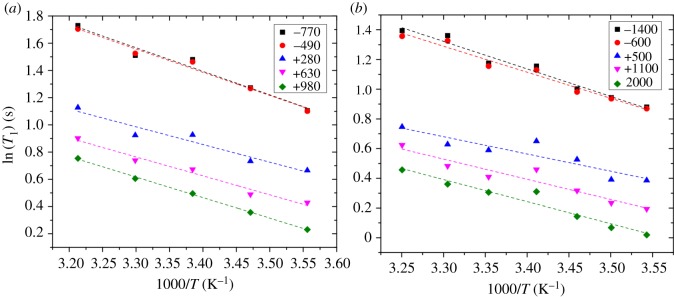
ln(*T*_1_) versus 1000/*T* for (*a*) bovine cartilage sample and (*b*) human cartilage sample. The legend in each case indicates which position in the *T*_1_ profiles (figure 2) the data were taken from. Negative numbers indicate data from above the cartilage surface (i.e. in the PBS) and positive numbers indicate data from within the cartilage tissue at different depths given in µm. Note that error bars in ln(*T*_1_) are not displayed as they are smaller than the size of the data points.

Figure 3 shows the Arrhenius plot for two data points selected at random in the PBS from each dataset in figure 2, and also three points from within the cartilage for each dataset, one from the upper regions, one in the middle and one towards the deeper regions of the cartilage. Figure 3 shows that the Arrhenius relationship is valid over the temperature range which has been studied, because the data show good linear dependence of ln(*T*_1_) on 1000/*T*. For all datasets visually inspected, the goodness of fit, *r*^2^, was greater than 0.8 in all cases. The Arrhenius model therefore adequately models the data.

### 3.3. *E*_A_ profiles

**Figure 4.**
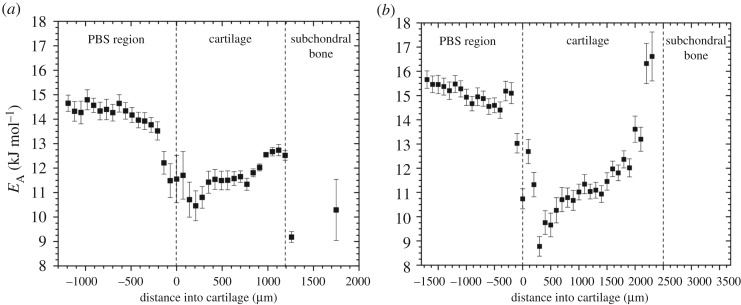
Activation energy profiles through the depth of the cartilage (*a*) bovine and (*b*) human. The PBS/cartilage/subchondral bone dividing lines are drawn to guide the eye and have been defined to be the same depths as those in figure 2, using the method described in that figure.

Figure 4 shows the depth-wise *E*_A_ profiles for the ROIs selected. A different trend from that observed for the *T*_1_ relaxation time depth-wise profiles in figure 2 is seen, which reveals that the activation energy profile is giving different and therefore additional information to that of the *T*_1_ profile; it is this that motivates this article. A detailed discussion of the nature and value of the additional information of the *E*_A_ is given in the conclusions. The *E*_A_ is lower in the cartilage than in the PBS, both in the human and bovine cartilage, and then again, in both cases, the activation energy increases in the deeper regions of the cartilage tissue. Similarly, the *T*_1_ is lower in the cartilage than in the PBS, but in contrast to *E*_A_, *T*_1_ continues to decrease with increasing depth into the cartilage. There are suggestions of other features in the profile: a local minimum, close to the surface of the cartilage and also a flattening of the *E*_A_ in the middle of the cartilage. It should be noted that the initial steep decrease in the *E*_A_ seen in figure 4, from the PBS to the cartilage, could be due to the same artefact discussed in the *T*_1_ profiles, a partial volume effect across the cartilage surface. Values within the subchondral bone are not all displayed, because the *E*_A_ values are unreliable and often negative in this region (see the paragraph above on the *T*_1_ values and issues within the subchondral bone).

*E*_A_ profiles for other cartilage samples imaged in this study are shown in the electronic supplementary material. Similar trends can be seen in four of the other samples imaged. There will be differences in biological constituents between the samples and hence also in *E*_A_. Additionally, an example of the fitted pre-factor from the Arrhenius model, *A*, for one sample is shown in the electronic supplementary material.

From data in Simpson & Carr [41], the spin–lattice *E*_A_ of pure water is 16.6 ± 0.4 kJ mol^−1^, and measurement of the spin–lattice *E*_A_ of the PBS in this study (measured separately by non-imaging NMR) was 17.0 ± 0.3 kJ mol^−1^, which are comparable to the values of *E*_A_ in the PBS in figure 4 of 14.7 ± 1.0 kJ mol^−1^.

The interesting result to note here is that the *E*_A_ of the water within the cartilage tissue (11 ± 2 kJ mol^−1^) is actually lower than that of the PBS (14.7 ± 1.0 kJ mol^−1^) or pure water (16.6 ± 0.4 kJ mol^−1^). We suggest that this is because the rotational dynamics of water is disrupted by the confinement of the water within the cartilage matrix. This is similar to how the confinement of water within pores lowers the melting and freezing point of water [42,43]. It has also been found for other systems that the diffusion activation energies of liquids are reduced by confinement into pores or channels, showing that the translational dynamics are affected by confinement [44]. Indeed, the lowering of the *E*_A_ in biological tissues below the value of pure water has previously been reported for relaxation times in muscle tissue [33] and diffusion in white matter [32]. Molecular dynamic simulations show that the confinement of water has a notable effect on the hydrogen bond network of water extending up to approximately 0.1 µm from a confining surface [45]. Neutron diffraction experiments have shown that confined water phases are radically different from free (unconfined) phases owing to the presence of cross-interactions between water and surface substrate, and that the perturbation induced by the wall extends throughout the confined region [46].

Diffusion, involving translational motion, will (in general) have a higher activation energy than one found from *T*_1_ or for rotational motion. According to the work of O'Reilly [47], the diffusion activation energy is the sum of rotational activation energy plus the work required to create a ‘vacancy’ for the diffusing molecule to move into. Our argument is that a lowering in diffusion activation energy caused by confinement, as reported in the literature, supports our explanation of a lowered activation energy for *T*_1_ in cartilage due to confinement. The fact that the absolute values will be different between the two different mechanisms does not detract from the point that the values are decreased by confinement.

We now suggest that the subsequent increase in *E*_A_ with depth is related to the association of the water to the other macromolecular constituents in the cartilage tissue, because it is well known that collagen density [4,48] and GAG density [4] increase with depth, while water density decreases [49]. There are, to a first approximation, effectively two types of water within cartilage tissue, relatively mobile ‘bulk’ water and relatively immobile ‘bound’ water that is associated with or bound to the macromolecules within the tissue. The bulk water has a reduced *E*_A_ because of confinement within the tissue pores as suggested above, and the bound water actually has a higher *E*_A_ because it is directly associated with or bound to macromolecules within the tissue. This additional binding increases the activation energy required for rotation, which subsequently increases the activation energy for spin–lattice relaxation. It should be noted that water that is confined within pores is not necessarily directly interacting with the confining surface, and hence is not necessarily part of the bound water in the sense described above. The *T*_1_ measurement, and thus the *E*_A_ value, is approximately a weighted average of these two types of water, and because the bulk water constitutes a greater fraction closer to the articular surface [49], the net effect is that *E*_A_ is lower than that of pure water near the surface. Deeper in the cartilage, this weighted average changes in favour of the bound water, because a greater fraction of the water is now directly associated with the macromolecules in this less water dense environment, and the measured *E*_A_ increases because of this.

## 4. Conclusion

Human and bovine cartilages were examined using MRI to measure the *T*_1_ in the tissue samples as a function of depth. This was repeated for a range of temperatures in order to determine the spin–lattice relaxation activation energy as a function of depth through the cartilage, using an Arrhenius model. Depth-wise *T*_1_ profiles through the cartilage matched values reported previously and showed similar trends through the depth of the tissues at all temperatures [26]. It was shown that the *T*_1_ relaxation time as a function of temperature, within the cartilage samples, follows Arrhenius-type behaviour. From this, an activation energy for the NMR relaxation process was determined. The activation energy within the cartilage (11 ± 2 kJ mol^−1^) is lower than that of pure water (16.6 ± 0.4 kJ mol^−1^) and PBS (14.7 ± 1.0 kJ mol^−1^). Profiles of the depth-wise activation energy were shown in figure 4, which show a different behaviour from those of the *T*_1_ relaxation time profiles, as shown in figure 3. The activation energy increases with depth, but is generally lower than that of pure water. This is to be compared with the *T*_1_ relaxation time which decreases with depth and is lower than that of pure water. This difference in behaviour indicates that the activation energy is giving additional information about the cartilage system.

It is important to note that the activation energy contains new and additional information to the spin–lattice relaxation time, *T*_1_. Though the Arrhenius equation links the spin–lattice relaxation time directly to the activation energy, it is not known *a priori*; without varying the temperature, it is not possible to determine the value of *E*_A_. It is the energy barrier that has to be overcome for *T*_1_ relaxation to occur. The *T*_1_ relaxation time at high fields (greater than 100 MHz) is determined predominantly by the rotational motion of, in this case, water molecules. Therefore, the NMR-determined *E*_A_ is effectively a measure of the rotational *E*_A_ of the water molecules. These water molecules exist in a variety of states, such as ‘free’ and ‘bound’ [30], but in our work only one spin–lattice relaxation time is evident. Hence, the *E*_A_ is a weighted average of the different environments and includes the effect of confinement on the arrangement of water through its effect on inter-water molecule interactions. This makes the *E*_A_ potentially interesting, as it is a measure of confinement and the interactions of the water molecules with other constituents within cartilage. Changes in composition of the cartilage, through ageing or damage, are therefore expected to alter the activation energy and hence it might prove to be a useful tool for cartilage (*in vitro*) research. As shown in figures 2 and 4, the dependence of the *E*_A_ on depth is different from that of the *T*_1_ relaxation time, confirming our hypothesis that the *E*_A_ reveals additional information to that of the *T*_1_ relaxation time.

Although further work is required to understand the physical meaning of *E*_A_, we suggest that the decrease in the activation energy, when compared with that for pure water, is due to the confinement of the water within the cartilage matrix, including by action of pores. Furthermore, we suggest that the subsequent increase of *E*_A_ with depth is due to the increase in association and bonding of the water with the other constituents deeper within the cartilage. This is because with increasing depth the water density itself decreases, while the density of other components increases [4], giving rise to more interactions between the water and those components. The increase of these interactions between the water and its surroundings leads to an increase in the activation energy for rotational motion of the water. If our hypothesis is correct, then this could make the activation energy a useful measure of the amount of bulk and bound water, and hence could be a novel way to detect structural changes, for instance those associated with OA, in cartilage (*in vitro*).

## Supplementary Material

Imaging of Nuclear Magnetic Resonance spin-lattice relaxation activation energy in cartilage: Supplementary Information

## Data accessibility

Data associated with this work are available from the Research Data Leeds repository under a CC-BY 4.0 licence at https://doi.org/10.5518/270. The code used for data analysis in this study is available from the Research Data Leeds repository under a GNU LGPL 3.0 licence at https://doi.org/10.5518/271.

## References

[1] Binks DA, Hodgson RJ, Ries ME, Foster RJ, Smye W, McGonagle D, Radjenovic A 2013 Quantitative parametric MRI of articular cartilage: a review of progress and open challenges. Br. J. Radiol. 86, 20120163 (10.1259/bjr.20120163) 23407427 PMC3608060

[2] Mow VC, Huiskes R 2005 Basic orthopaedic biomechanics and mechano-biology, 3rd edn Philadelphia, PA: Lippincott Williams and Wilkins.

[3] Vaca-González JJ, Guevara JM, Moncayo MA, Castro-Abril H, Hata Y, Garzón-Alvarado DA 2017 Biophysical stimuli: a review of electrical and mechanical stimulation in hyaline cartilage. Cartilage (online).(doi:10.1177/1947603517730637) 10.1177/1947603517730637 PMC6425540 28933195

[4] Wilson W, van Donkelaar CC, van Rietbergen R, Huiskes R 2005 The role of computational models in the search for the mechanical behavior and damage mechanisms of articular cartilage. Med. Eng. Phys. 27, 810–826. (10.1016/j.medengphy.2005.03.004) 16287601

[5] Pacifici M 2014 Introduction to the mini-review series ‘Articular cartilage: biology, pathology and repair’. Matrix. Biol. 39, 1 (10.1016/j.matbio.2014.08.004) 25172827

[6] McGonagle D, Tan AL, Carey J, Benjamin M 2010 The anatomical basis for a novel classification of osteoarthritis and allied disorders. J. Anat. 216, 279–291. (10.1111/j.1469-7580.2009.01186.x) 20070426 PMC2829386

[7] Baldassarri M, Goodwin JSL, Farley ML, Bierbaum BE, Goldring SR, Goldring MB, Burstein D, Gray ML 2007 Relationship between cartilage stiffness and dGEMRIC index: correlation and prediction. J. Orthop. Res. 25, 904–912. (10.1002/jor.20378) 17427215

[8] Nieminen MT, Rieppo J, Toyras J, Hakumaki JM, Silvennoinen J, Hyttinen MM, Helminen HJ, Jurvelin JS 2001 T-2 relaxation reveals spatial collagen architecture in articular cartilage: a comparative quantitative MRI and polarized light microscopic study. Magn. Reson. Med. 46, 487–493. (10.1002/mrm.1218) 11550240

[9] Nissi MJ, Rieppo J, Toyras J, Laasanen MS, Kiviranta I, Jurvelin JS, Nieminen MT 2006 T-2 relaxation time mapping reveals age- and species-related diversity of collagen network architecture in articular cartilage. Osteoarthr. Cartil. 14, 1265–1271. (10.1016/j.joca.2006.06.002) 16843689

[10] Xia Y, Moody JB, Alhadlaq H 2002 Orientational dependence of *T*_2_ relaxation in articular cartilage: a microscopic MRI (μMRI) study. Magn. Reson. Med. 48, 460–469. (10.1002/mrm.10216) 12210910

[11] Bashir A, Gray ML, Hartke J, Burstein D 1999 Nondestructive imaging of human cartilage glycosaminoglycan concentration by MRI. Magn. Reson. Med. 41, 857–865. (10.1002/(SICI)1522-2594(199905)41:5%3C857::AID-MRM1%3E3.0.CO;2-E) 10332865

[12] Dunn TC, Lu Y, Jin H, Ries MD, Majumdar S 2004 T2 relaxation time of cartilage at MR imaging: comparison with severity of knee osteoarthritis. Radiology 232, 592–598. (10.1148/radiol.2322030976) 15215540 PMC4447089

[13] Eckstein F, Burstein D, Link TM 2006 Quantitative MRI of cartilage and bone: degenerative changes in osteoarthritis. NMR Biomed. 19, 822–854. (10.1002/nbm.1063) 17075958

[14] Zarins ZA, Bolbos RI, Pialat JB, Link TM, Li X, Souza RB, Majumdar S 2010 Cartilage and meniscus assessment using T1rho and T2 measurements in healthy subjects and patients with osteoarthritis. Osteoarthr. Cartil. 18, 1408–1416. (10.1016/j.joca.2010.07.012) 20696262 PMC2975868

[15] Fragonas E, Mlynárik V, Jellús V, Micali F, Piras A, Toffanin R, Rizzo R, Vittur F 1998 Correlation between biochemical composition and magnetic resonance appearance of articular cartilage. Osteoarthr. Cartil. 6, 24–32. (10.1053/joca.1997.0089) 9616436

[16] Xia Y, Moody JB, Burton-Wurster N, Lust G 2001 Quantitative in situ correlation between microscopic MRI and polarized light microscopy studies of articular cartilage. Osteoarthr. Cartil. 9, 393–406. (10.1053/joca.2000.0405) 11467887

[17] Rautiainen J et al. 2015 Multiparametric MRI assessment of human articular cartilage degeneration: correlation with quantitative histology and mechanical properties. Magn. Reson. Med. 74, 249–259. (10.1002/mrm.25401) 25104181 PMC4320684

[18] Lüssea S, Claassen H, Gehrke T, Hassenpflug J, Schünke M, Heller M, GlüEr C-C 2000 Evaluation of water content by spatially resolved transverse relaxation times of human articular cartilage. Magn. Reson. Imaging 18, 423–430. (10.1016/S0730-725X(99)00144-7) 10788720

[19] Akella SVS, Regatte RR, Gougoutas AJ, Borthakur A, Shapiro EM, Kneeland JB, Leigh JS, Reddy R 2001 Proteoglycan-induced changes in T_1ρ_-relaxation of articular cartilage at 4 T. Magn. Reson. Med. 46, 419–423. (10.1002/mrm.1208) 11550230

[20] Wheaton AJ, Dodge GR, Elliott DM, Nicoll SB, Reddy R 2005 Quantification of cartilage biomechanical and biochemical properties via *T*_1ρ_ magnetic resonance imaging. Magn. Reson. Med. 54, 1087–1093. (10.1002/mrm.20678) 16200568

[21] Borthakur A, Mellon E, Niyogi S, Witschey W, Kneeland JB, Reddy R 2006 Sodium and *T*_1ρ_ MRI for molecular and diagnostic imaging of articular cartilage. NMR Biomed. 19, 781–821. (10.1002/nbm.1102) 17075961 PMC2896046

[22] Reddy R, Insko EK, Noyszewski EA, Dandora R, Kneeland JB, Leigh JS 1998 Sodium MRI of human articular cartilage *in vivo*. Magn. Reson. Med. 39, 697–701. (10.1002/mrm.1910390505) 9581599

[23] Shapiro EM, Borthakur A, Gougoutas A, Reddy R 2002 _23_Na MRI accurately measures fixed charge density in articular cartilage. Magn. Reson. Med. 47, 284–291. (10.1002/mrm.10054) 11810671 PMC2858596

[24] Lin PC, Reiter DA, Spencer RG 2009 Sensitivity and specificity of univariate MRI analysis of experimentally degraded cartilage. Magn. Reson. Med. 62, 1311–1318. (10.1002/mrm.22110) 19705467 PMC2966866

[25] Ward KM, Aletras AH, Balaban RS 2000 A new class of contrast agents for MRI based on proton chemical exchange dependent saturation transfer (CEST). J. Magn. Reson. 143, 79–87. (10.1006/jmre.1999.1956) 10698648

[26] Nissi MJ, Rieppo J, Töyräs J, Laasanen MS, Kiviranta I, Nieminen MT, Jurvelin JS 2007 Estimation of mechanical properties of articular cartilage with MRI – dGEMRIC, *T*_2_ and *T*_1_ imaging in different species with variable stages of maturation. Osteoarthr. Cartil. 15, 1141–1148. (10.1016/j.joca.2007.03.018) 17513137

[27] Berberat JE, Nissi MJ, Jurvelin JS, Nieminen MT 2009 Assessment of interstitial water content of articular cartilage with *T*_1_ relaxation. Magn. Reson. Imaging 27, 727–732. (10.1016/j.mri.2008.09.005) 19056195

[28] Burstein D, Velyvis J, Scott KT, Stock KW, Kim YJ, Jaramillo D, Boutin RD, Gray ML 2001 Protocol issues for delayed Gd(DTPA)-enhanced MRI: (dGEMRIC) for clinical evaluation of articular cartilage. Magn. Reson. Med. 45, 36–41. (10.1002/1522-2594(200101)45:1%3C36::AID-MRM1006%3E3.0.CO;2-W) 11146483

[29] Tiderius CJ, Olsson LE, Leander P, Ekberg O, Dahlberg L 2003 Delayed gadolinium-enhanced MRI of cartilage (dGEMRIC) in early knee osteoarthritis. Magn. Reson. Med. 49, 488–492. (10.1002/mrm.10389) 12594751

[30] Damion RA, Pawaskar SS, Ries ME, Ingham E, Williams S, Jin Z, Radjenovic A 2012 Spin–lattice relaxation rates and water content of freeze-dried articular cartilage. Osteoarthr. Cartil. 20, 184–190. (10.1016/j.joca.2011.12.005) 22197886

[31] Rieke V, Butts Pauly K, Pauly KB 2008 MR thermometry. J. Magn. Reson. Imaging 27, 376–390. (10.1002/jmri.21265) 18219673 PMC2780364

[32] Dhital B, Labadie C, Möller HE, Turner R 2011 Activation energies for water diffusion in ex-vivo white matter vol. 19. In Proceedings of the International Society for Magnetic Resonance in Medicine, Montreal, Canada, 613 May p. 78 ISMRM. See https://www.ismrm.org/11/Session08.htm.

[33] Fung BM, McGaughy TW 1979 Study of spin-lattice and spin-spin relaxation-times of 1H, 2H, and 17O in muscle water. Biophys. J. 28, 293–303. (10.1016/S0006-3495(79)85177-2) 233613 PMC1328631

[34] Quesson B, de Zwart JA, Moonen CTW 2000 Magnetic resonance temperature imaging for guidance of thermotherapy. J. Magn. Reson. Imaging 12, 525–533. (10.1002/1522-2586(200010)12:4%3C525::AID-JMRI3%3E3.0.CO;2-V) 11042633

[35] Wright A 2014 Leeds tissue bank: making a GIFT for research Vol. 2014. Leeds, UK: University of Leeds.

[36] Cline HE, Hynynen K, Hardy CJ, Watkins RD, Schenck JF, Jolesz FA 1994 MR temperature mapping of focused ultrasound surgery. Magn. Reson. Med. 31, 628–636. (10.1002/mrm.1910310608) 8057815

[37] Matsumoto R, Oshio K, Jolesz FA 1992 Monitoring of laser and freezinginduced ablation in the liver with T1-weighted MR imaging. J. Magn. Reson. Imaging 2, 555–562. (10.1002/jmri.1880020513) 1392248

[38] Hynynen K, McDannold N, Mulkern RV, Jolesz FA 2000 Temperature monitoring in fat with MRI. Magn. Reson. Med. 43, 901–904. (10.1002/1522-2594(200006)43:6%3C901::AID-MRM18%3E3.0.CO;2-A) 10861887

[39] Horch RA, Nyman JS, Gochberg DF, Dortch RD, Does MD 2010 Characterization of ^1^H NMR signal in human cortical bone for magnetic resonance imaging. Magn. Reson. Med. 64, 680–687. (10.1002/mrm.22459) 20806375 PMC2933073

[40] Karampinos DC, Melkus G, Baum T, Bauer JS, Rummeny EJ, Krug R 2014 Bone marrow fat quantification in the presence of trabecular bone: initial comparison between water-fat imaging and single-voxel MRS. Magn. Reson. Med. 71, 1158–1165. (10.1002/mrm.24775) 23657998 PMC3759615

[41] Simpson JH, Carr HY 1958 Diffusion and nuclear spin relaxation in water. Phys. Rev. 111, 1201 (10.1103/PhysRev.111.1201)

[42] Babushkina TA, Klimova TP, Shtykova AV, Dembo KA, Volkov VV, Khripunov AK, Klechkovskaya VV 2010 Study of the gel films of *Acetobacter xylinum* cellulose and its modified samples by ^1^H NMR cryoporometry and small-angle X-ray scattering. Crystallogr. Rep. 55, 312–317. (10.1134/S1063774510020252)

[43] Hay JN, Laity PR 2000 Observations of water migration during thermoporometry studies of cellulose films. Polymer 41, 6171–6180. (10.1016/S0032-3861(99)00828-9)

[44] Lingwood MD, Zhang ZY, Kidd BE, McCreary KB, Hou JB, Madsen LA 2013 Unraveling the local energetics of transport in a polymer ion conductor. Chem. Commun. 49, 4283–4285. (10.1039/c2cc37173a) 23282487

[45] Fan Y, Gao YQ 2010 Long-range effects of confinement on water structure. J. Phys. Chem. B 114, 4246–4251. (10.1021/jp9086392) 20210292

[46] Mancinelli R 2010 The effect of confinement on water structure. J. Phys. Condens. Matter 22, 404213 (10.1088/0953-8984/22/40/404213) 21386574

[47] O'Reilly DE 1968 Self-diffusion coefficients and rotational correlation times in polar liquids. J. Chem. Phys. 49, 5416–5420. (10.1063/1.1670066)

[48] Kokkonen HT et al. 2011 Computed tomography detects changes in contrast agent diffusion after collagen cross-linking typical to natural aging of articular cartilage. Osteoarthr. Cartil. 19, 1190–1198. (10.1016/j.joca.2011.07.008) 21827864

[49] Wilson W, Huyghe JM, van Donkelaar CC 2007 Depth-dependent compressive equilibrium properties of articular cartilage explained by its composition. Biomech. Model. Mechanobiol. 6, 43–53. (10.1007/s10237-006-0044-z) 16710737

