# Supplementary material for: Imaging of nuclear magnetic resonance spin–lattice relaxation activation energy in cartilage
Source: R Soc Open Sci. 2018 Jul 11;5(7):180221. doi: 10.1098/rsos.180221 (PMC6083713; doi:10.1098/rsos.180221)
Supplement: Imaging of Nuclear Magnetic Resonance spin-lattice relaxation activation energy in cartilage: Supplementary Information [file rsos180221supp1.pdf]

Imaging of Nuclear Magnetic Resonance spin-lattice relaxation activation energy in cartilage

R.J. Foster, R.A. Damion, M.E. Ries, S.W. Smye, D.G. McGonagle, D.A. Binks, A. Radjenovic

Supplementary Information

This supplementary information is associated with the publication of the same title in Royal Society: Open Science.

Sample Details

Note that only relevant medical history related to musculoskeletal diagnoses has been included

**Sample**	**Identifier**	**Type**	**Details**	**Results Presented**
1	G18_11	Human	86 year old female.	Main manuscript
2	Bovine	Bovine	Approx 18month old cow. Harvested approximately within 72hours of slaughter.	Main manuscript
3	G05_11	Human	67year old female, Osteomyelitis	Supplementary Information
4	G24_09	Human	65 year old female, Spinal degeneration.	Supplementary Information
5	G29_10R	Human	66 year old male.	Supplementary Information
6	G48_11R Med	Human	70 year old male	Supplementary Information
7	G48_11R Lat	Human	70 year old male	Supplementary Information

Imaging Details

Sample	Repetition times (ms)	Temperatures (°C)*	Matrix Size	Resolution (μ/pixel)
1	200, 500, 1000, 1500, 4000, 8000	9.1, 12.5, 15.8, 20.0, 25.0, 29.4, 34.5	434x256	100x100
2	Varied at different temperatures – see below		128x256	70x117
3	200, 500, 1000, 1500, 2500, 4000, 8000	7.1, 12.6, 17.5, 21.5, 27.7, 34.3, 38.8	225x355	70x70
4	200, 500, 1000, 1500, 3000, 5000	10.5, 21.0, 26.9, 30.5, 38.0**	256x256	170x170
5	200, 500, 1000, 1500, 3000, 5000	7.8, 15.7, 22.0, 29.1, 36.9,	185x330	70x70
6	200, 500, 1000, 1500, 3000, 5000	7.7, 15.3, 20.9, 26.0 , 36.3	120x270	100x100
7	110, 200, 300, 500, 1000, 1500, 2000, 4000, 8000, 16000	9.1, 17.0, 24.2, 33.3, 37.8	171x342	70x70

*For all samples (except #4 – see note below), the temperature was monitored by a thermocouple within the bore of the magnet, using a standard Bruker NMR configuration. The temperature was automatically monitored during the whole imaging sequence and the temperature of imaging was taking as the (arithmetically fitted) average during the imaging sequence. The error in all temperature measurements is less than ±0.1°C. When changing the temperature, samples were given a minimum of 1 hour to equilibrate at the new temperature before starting the imaging sequence.

**There was a problem with the monitoring thermocouple when imaging this sample. Temperatures were taken as the user observed temperature whilst the imaging sequence was running. The error in these temperature measurements therefore is approximately ±1°C.

Bovine Cartilage (sample 2) imaging details

Sample	Temperature (°C)	T_R (ms)	Averages
Bovine	8.1	295, 458, 634, 824, 1033, 1263, 1519, 1808, 2139, 2528, 2999, 3595, 4407, 5692, 9000	4
	14.9	100, 287, 487, 703, 937, 1193, 1475, 1790, 2144, 2551, 3029, 3608, 4340, 5340, 6924, 11040	1
	22.3	213, 440, 684, 946, 1231, 1543, 1886, 2268, 2699, 3195, 3776, 4478, 5368, 6582, 8500, 13440	1
	30.0	100, 370, 660, 970, 1310, 1680, 2090, 2550, 3060, 3650, 4350, 5190, 6250, 7700, 10000, 16000	2
	38.1	622, 966, 1338, 1740, 2181, 2666, 3206, 3816, 4516, 5337, 6331, 7589, 9304, 12017, 19000	2

T1 fitting

A number of pixels were chosen at random throughout the cartilage to investigate whether a mono-exponential T1 fit was suitable. Mono-exponential fits were a good fit for all data, with an r^2 of greater than 0.99 in all cases within the cartilage tissue.

Figure I shows T1 fits for the same pixel at each temperature imaged. Figure II shows a series of pixels chosen at random from within the cartilage body in sample #3 at 21°C.

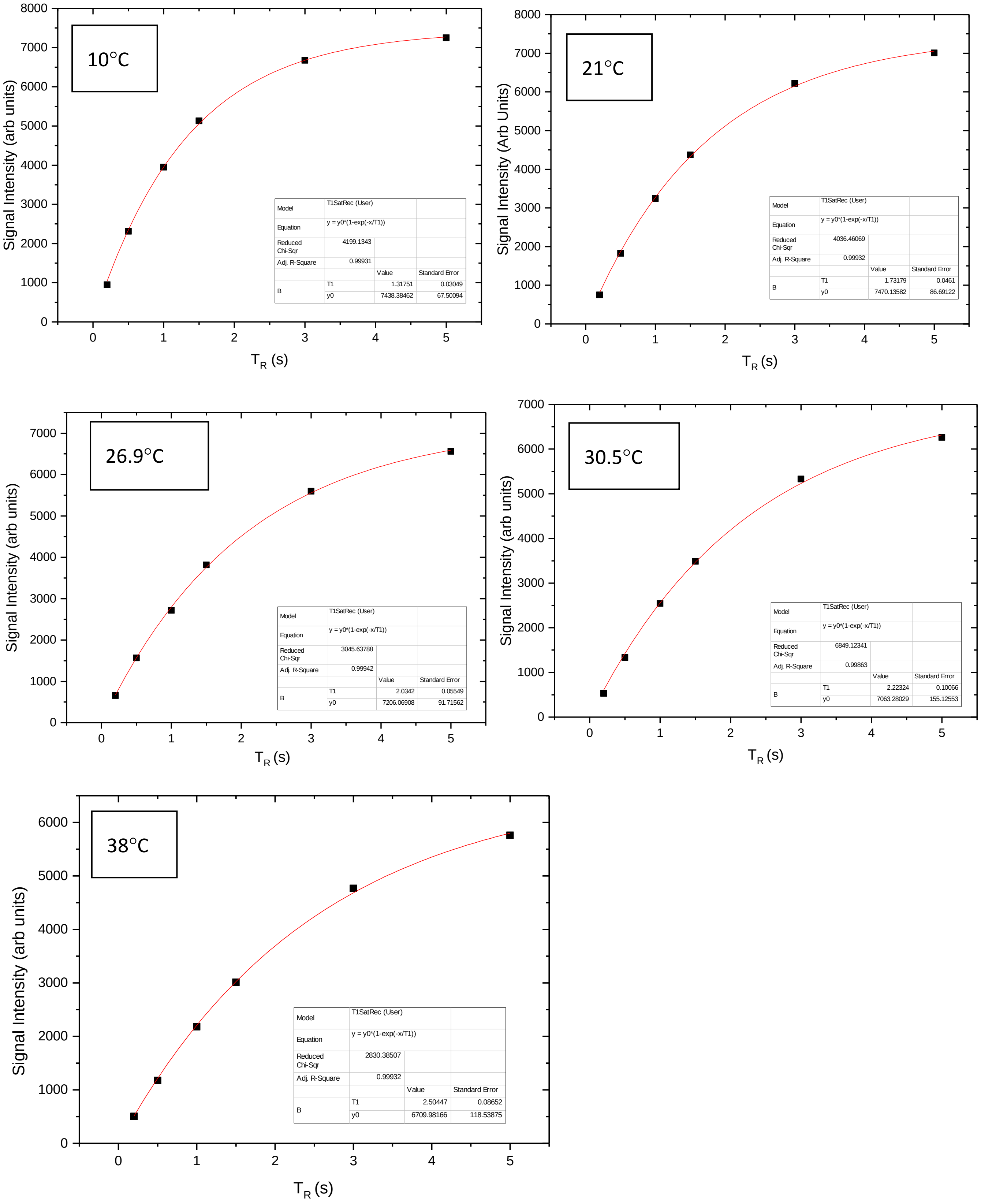

Figure I. T1 fits for the same pixel at different temperatures. Sample #4 (G24_09).

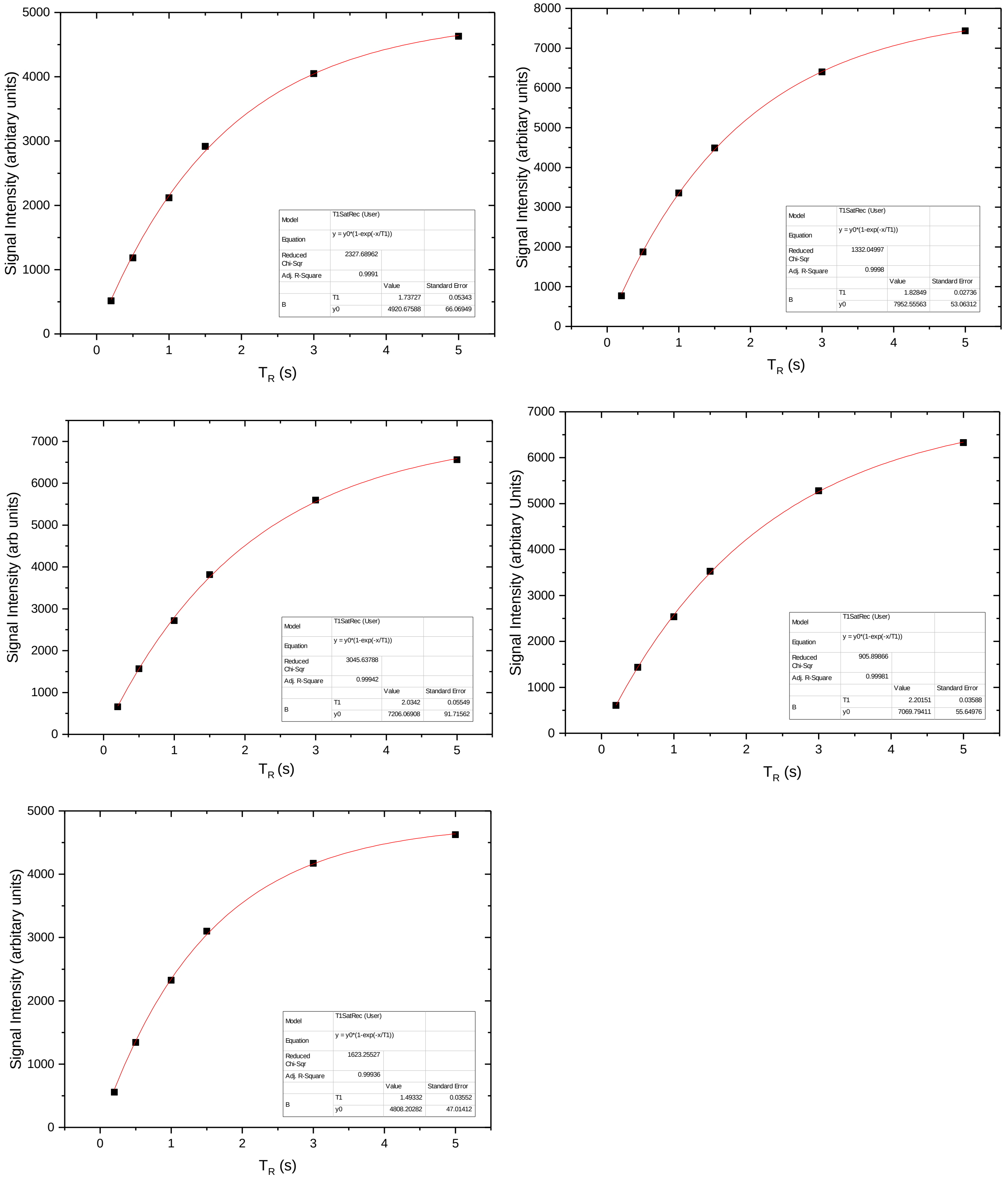

Figure II T1 fits for a series of pixels chosen at random from within the cartilage at different depths. Imaging at 26.9°C. Sample #4 (G24_09).

T1 and EA profiles for samples #3-#7

Error bars in T1 are smaller than the size of data points. Samples #1 and #2 are presented in the main manuscript.

For all samples, a slice in the imaging sequence close to the centre of cartilage has been used for E_A analysis. For samples #1 - #3 and #5 - #7, this was either slice 4 or 5, in the centre of the imaging package (the exact number depended on the total number of slices used, which varied due to the physical size of the sample). These were chosen so that the slice used for analysis was well away from any effects close to the edge of the sample. The slice number used for each sample is indicated below. For sample #4, only four slices were imaged, but due to the relative larger sample size, all slices were close to the centre of the cartilage. Therefore slice 1 was chosen for analysis in this sample.

Note that the dotted lines are estimated delineations between the PBS and cartilage, and in especially between the cartilage and subchondral bone. A description of how these were estimated is given in the figure caption for Figure 2 in the main manuscript.

Samples #2, #4, #5 and #7 show small changes in T1 with position within the PBS at the highest temperatures, and sample #3 shows a greater scatter in the data at these temperatures. This is due to the fact that the samples were immersed in PBS, but due to the relative size within the imaging tube in the NMR, the surface of the PBS generated artefacts visible in the PBS at higher temperatures. Although the region of interest was chosen carefully to avoid these artefacts, there may have been some segments in the PBS that were affected. In addition, it is possible that there are some edge effects or partial volume effects at the PBS/cartilage interface. Care was taken in the choice of imaging parameters to minimise these artefacts, but it was not possible to completely remove them because the parameters were determined in order to obtain the best T1 data throughout the cartilage tissue.

However, we note that the change in T1 with position at the higher temperatures is small compared to the change in T1 with temperature, meaning that the effect on EA is small. The Arrhenius fits gave a goodness of fit, r^2 of greater than 0.8 in all the random datasets sampled for visual checking of the fits.

Sample #3 (G05_11)

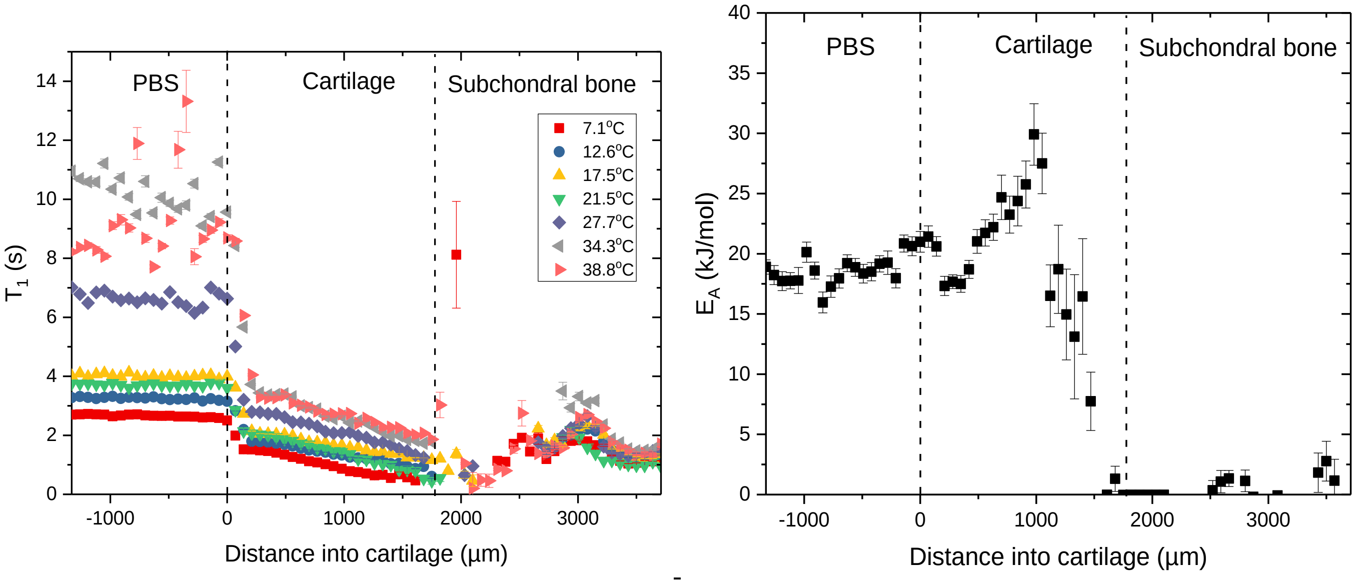

Figure III T1 and EA profiles for slice #4 of sample #3 (G05_11)

Sample #4 (G24_09)

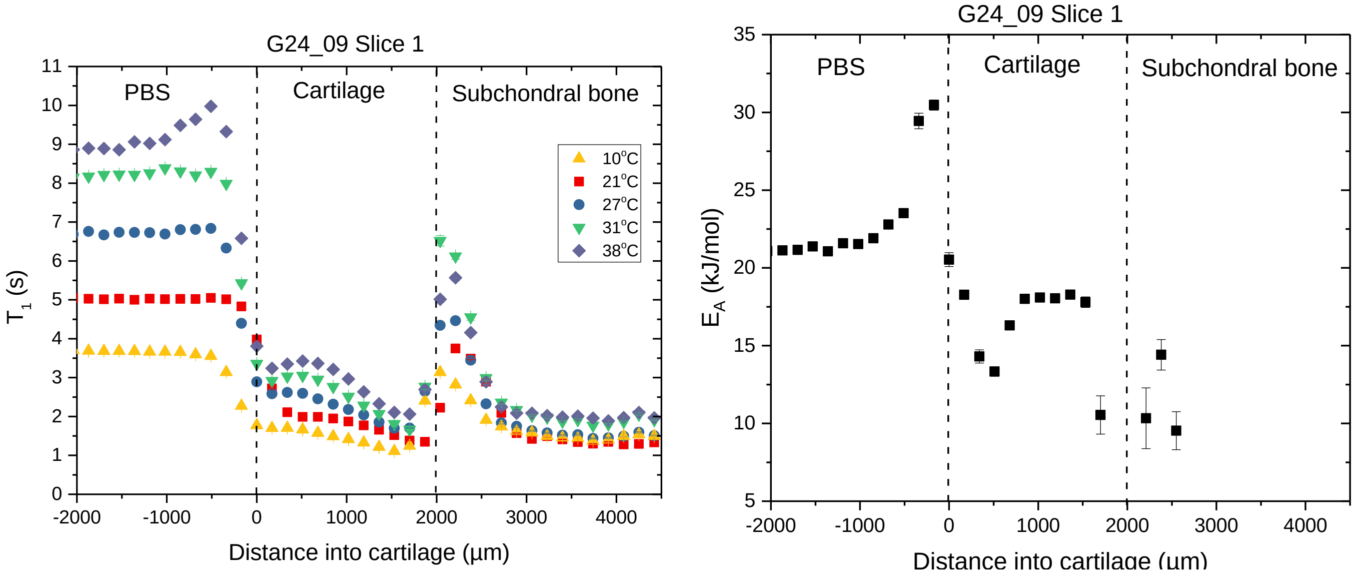

Figure IV T1 and EA profiles for slice #1 of sample #4 (G24_09)

Sample #5 (G29_10R)

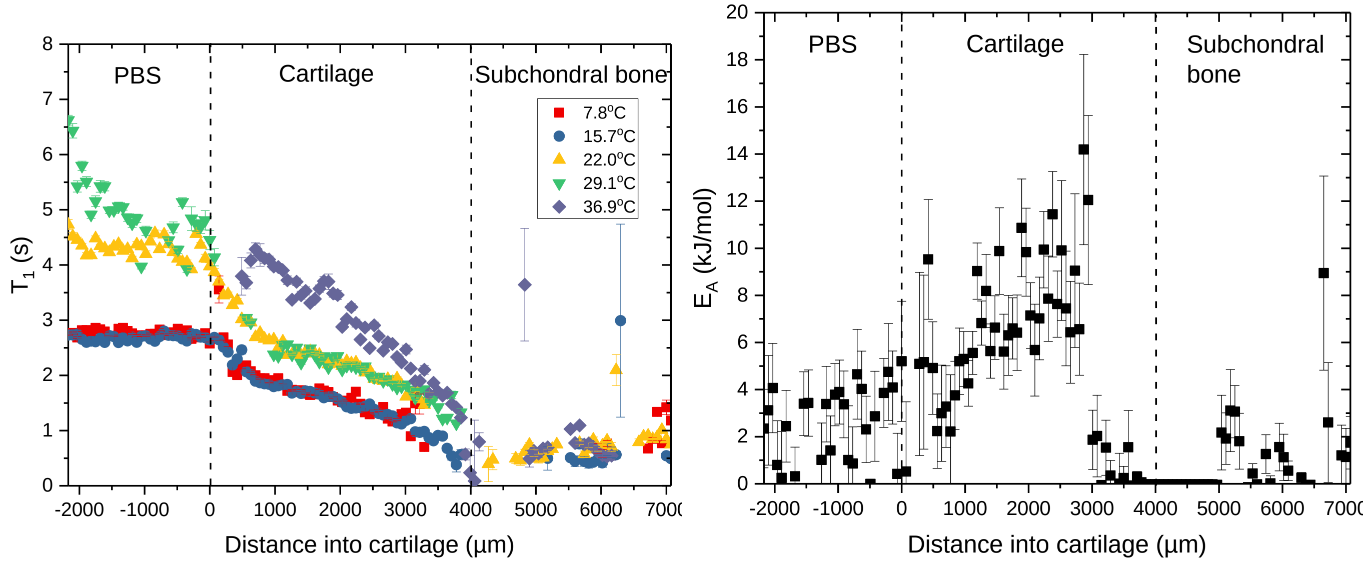

Figure V T1 and EA profiles for slice #4 of sample #5 (G29_10R)

Sample #6 (G48_11R Med)

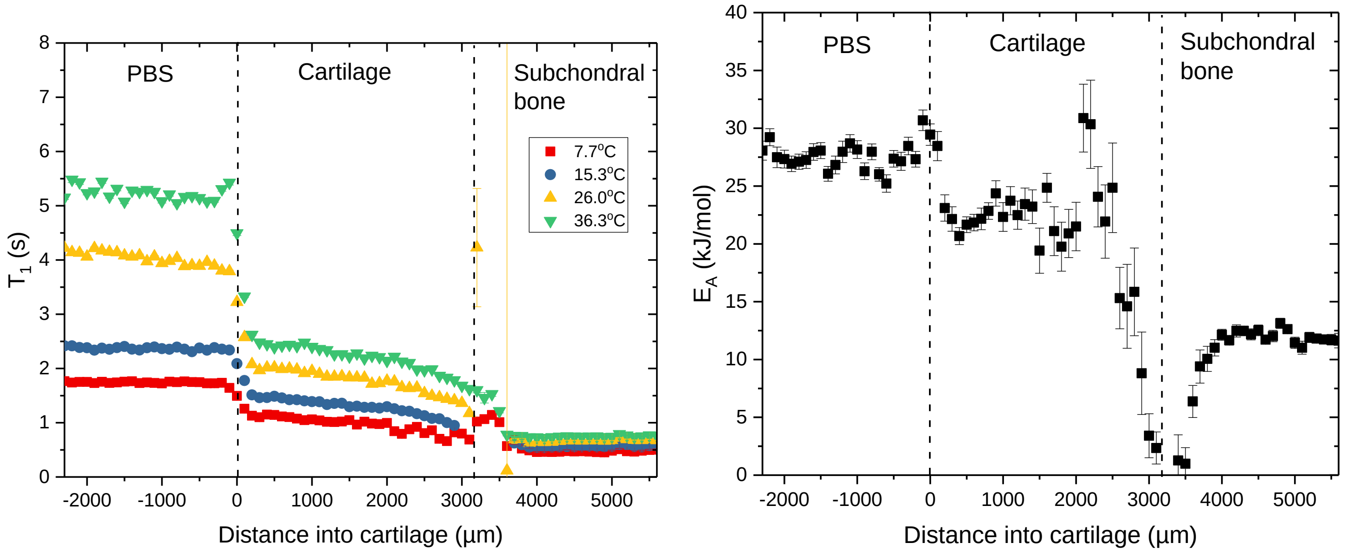

Figure VI T1 and EA profiles for slice #4 of sample #6 (G48_11R Med)

Sample #6 (G48_11RMed) moved during imaging after the T1 imaging data was obtained at 20.9°C (NB: this imaging dataset was the first collected). This dataset was therefore omitted when calculating EA profiles for this sample. The sample did not move further during the imaging session at other temperatures.

Sample #6 was fitted from DICOM images of the datasets.

<u>Sample #7 (G48_11R Lat)</u>

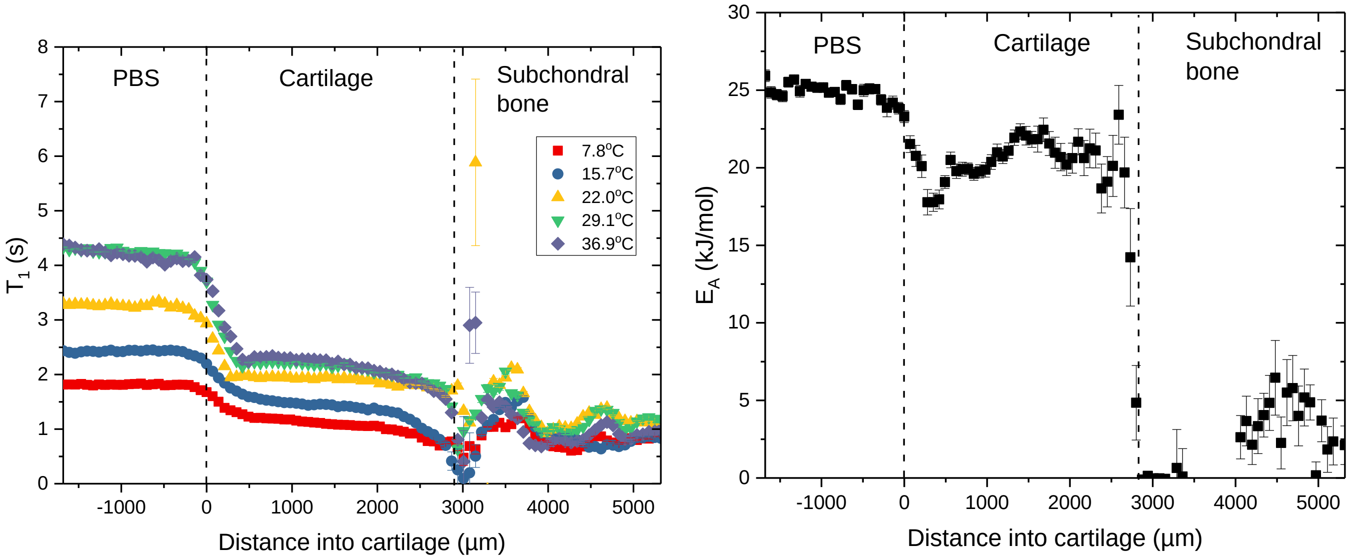

Figure VII T1 and EA profiles for slice #3 of sample #6 (G48_11R Lat)

Due to a user error, the datasets for T1 imaging at 9.1 and 17.0°C for sample #7 (G48_11RLat) were obtained with the slice packages rotated through 180° in the axial direction. The x and y positions of the slices remained the same. Upon post-processing, theT1-fitted data for each slice in the datasets at 9.1 and 17.0°C were mirrored to return them to the same orientation as the datasets obtained at the other temperatures. Note that because the slice packages were rotated by 180°, the slice order is reversed for 9.1 and 17.0°C datasets.

Sample #7 was fitted from DICOM images of the datasets.

<u>Exponential pre-factor analysis</u>

The Arrhenius plots shown in Figure 3 also allow the exponential pre-factor, *A*, from the Arrhenius model to be determined from the fitted intercepts. Figure VIII shows an example dataset of how T1 at 20°C, EA and A vary with depth through the cartilage for sample #1 (G18_11).

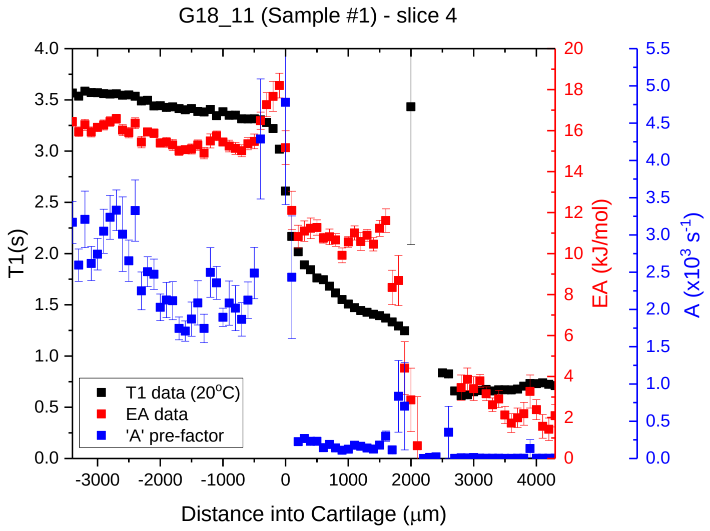

Figure VIII T1, EA and exponential pre-factor, A, as a function of depth for slice #4 of sample #1 (G18_11)

The pre-factor, A, can be related to a rotational correlation time and this is an extrapolation into the inertial limit of a completely free rotor model (Carper et al, J PhysChem, 100 (1996) p4724). It must be noted though that there is an entropic term within A that is usually not considered in an Arrhenius type analysis.

Data and Code Accessibility

Data associated with this work are available from the Research Data Leeds repository under a CC-BY 4.0 license at https://doi.org/10.5518/270.

The data for samples #1-#5 is uploaded in raw binary format taken directly from the Bruker NMR (Paravision). The data for samples #6 and #7 is uploaded in DICOM format. These were converted from the raw binary data using Paravision 5.1 (Bruker NMR).

Data for the figures plotted both in the main manuscript and the supplementary information is also uploaded to the same location in a single Excel spreadsheet with tabs labelled for each figure.

The code used for data analysis in this study is available from the Research Data Leeds repository under a GNU LGPL 3.0 license at https://doi.org/10.5518/271.

All code files written by the authors for this study (RAD and RJF) are included in the package. All code was written in Matlab R2013a. Open-access third-party code used as part of the analysis is not included in the package, but the code files used are documented within the files.
